# Effect of clonidine and magnesium sulphate on anaesthetic consumption, haemodynamics and postoperative recovery: A comparative study

**DOI:** 10.4103/0019-5049.63659

**Published:** 2010

**Authors:** Manjushree Ray, Dhurjoti Prosad Bhattacharjee, Bimal Hajra, Rita Pal, Nilay Chatterjee

**Affiliations:** Department of Anaesthesiology, N.R.S. Medical College, Kolkata, India; 1 Department of Anaesthesiology, Calcutta National Medical College, Kolkata, India; 2 Department of Calcutta Medical Research Institute, Kolkata, India

**Keywords:** Anaesthetic consumption, bispectral index, clonidine, magnesium sulphate

## Abstract

This randomised, placebo-controlled, double-blind study was designed to assess the effect of intravenous clonidine and magnesium sulphate on intraoperative haemodynamics, anaesthetic consumption and postoperative recovery. Seventy five patients undergoing elective upper limb orthopaedic surgery were randomised into three groups. Group C received clonidine 3 μg/kg as a bolus before induction and 1μg/kg/hour by infusion intraopertively. Group M received magnesium sulphate 30 mg/kg as a bolus before induction and 10 mg/kg/hour by infusion. Group P received same volume of isotonic saline. Anaesthesia was induced and maintained with fentanyl citrate and propofol. Muscular relaxation was achieved by vecuronium bromide. Induction time, recovery time and consumption of propofol as well as fentanyl citrate were recorded. Induction of anaesthesia was rapid with both clonidine and magnesium sulphate. Time of bispectral index (BIS) to reach 60 was significantly lower in Group C and Group M (*P* < 0.0001). Requirements of propofol and fentanyl were significantly less in Group C and Group M (*P* < 0.001). Postoperative recovery was slower in Group M compared with other two groups (*P* < 0.001). Perioperative use of both clonidine and magnesium sulphate significantly reduced the consumption of propofol and fentanyl citrate. Magnesium sulphate caused a delayed recovery.

## INTRODUCTION

Clonidine is an imidazoline derivative with alpha 2 agonistic activity. It has intrinsic analgesic effect and produces preoperative sedation and anxiolysis. The potential advantages of using clonidine during anaesthesia are improved intraoperative haemodynamic stability, attenuated sympathoadrenal responses to laryngoscopy and tracheal intubation, reduced intraoperative requirement of anaesthetic agents and reduced postoperative pain.[1]

Parenteral magnesium sulphate (MgSO_4_) has been used for many years as an antiarrhythmic agent and for prophylaxis against seizures in pre-eclampsia. Recently, the importance of magnesium in anaesthetic practice has been highlighted.[2] It has been suggested that magnesium (Mg) has got the potential to treat and prevent pain by acting as an antagonist of *N*-methyl *D*-aspartate (NMDA) receptors.[3,4] In animals, Mg suppressed NMDA-induced adverse behavioral reactions and hypersensitivities resulting from nerve injuries. In a clinical study, the role of Mg in reducing analgesic requirements during the postoperative period has been demonstrated.[5] Koinig and colleagues[6] reported that Mg administration led to a significant reduction in fentanyl consumption in the peri and postoperative periods.

This placebo-controlled, double-blind prospective study is designed to assess the effect of intravenous clonidine and MgSO_4_ on intraoperative haemodynamics, anaesthetic consumption and postoperative recovery, when they are used as adjunctive agents.

## METHODS

The study protocol was approved by the Institutional Ethical Committee of N.R.S. Medical College, Kolkata, and informed consent was taken from each of the patients. Seventy five ASA Grade I and II patients undergoing elective upper limb orthopaedic surgery were randomly assigned to one of the three following groups: Group C (clonidine group), Group M (magnesium group) and Group P (control group). Exclusion criterion included hypertension, morbid obesity, alcohol/drug abuse, hepatic, renal, endocrine and cardiac dysfunction.

On arrival to the operation theatre, monitors were attached and baseline vital parameters like heart rate, mean arterial blood pressure (MAP), and arterial oxygen saturation (SpO_2_) were recorded. An intravenous line was started. The level of anaesthesia was monitored with bispectral index (BIS^TM^). The BIS electrodes were placed on the forehead and on the lateral angle of orbit and connected to A-2000 BIS monitoring system. The target BIS range was 40–60 for surgical anaesthesia.[7] For Group C patients, 600 *μ*g clonidine (4 ml) was diluted with 16 ml distilled water in a 20-ml syringe(1 ml = 30 *μ*g). Similarly, 8 ml distilled water was added to 12 ml magnesium sulphate (6000 mg) in a 20-ml syringe for Group M patients (1 ml = 300 mg). Normal saline was used for Group P patients. The solutions were prepared by an anaesthesiologist, who was totally unaware of nature of the study. Patients received 0.1 ml/kg of one of these above-said solutions as a bolus over a period of 15 minutes before induction. Same solution was administered at a rate of 0.03 ml/kg/hour as continuous intravenous infusion throughout the surgical procedure.

After preoxygenation for 3 minutes, patients received 1 *μ*g/kg of fentanyl citrate intravenously. They were induced by injection propofol, administered at a rate of 20 mg per 5 seconds until BIS was below 60. Endotracheal intubation was facilitated by muscle relaxant vecuronium bromide. Anaesthesia was maintained by nitrous oxide and oxygen (50% + 50%) along with propofol infusion, started at the rate of 10 mg/kg/hour and titrated to maintain a BIS in the range of 40–60. Dose adjustment of fentanyl was based on standard clinical signs and haemodynamic measurements. Signs of inadequate analgesia, defined as an increase of heart rate and MAP of more than 20% of baseline, were to be managed by a bolus dose of fentanyl 0.5 *μ*g/kg [if BIS was within the recommended range (40–60)].[6] Muscle relaxation was achieved by intermittent bolus doses of vecuronium bromide. The patients were mechanically ventilated to keep EtCO_2_ between 35 and 40 mm Hg. Normothermia was maintained during the operation.

At the end of the surgical procedure, all the infusions were stopped. Time for BIS to rise to 80 was recorded. Residual neuromuscular block was reversed by an appropriate dose of neostigmine and glycopyrrolate. Tracheal extubation was performed and the following times were noted down: (i) time to tracheal extubation, (ii) time to response to verbal commands (spontaneous eye opening), (iii) orientation time (to recollect name, date of birth and location). Recovery time was measured by using the time to respond to verbal command and orientation time. Heart rate, MAP, SpO_2_ were also recorded throughout the surgical procedure at an interval of 10 minutes. The patients were observed for any adverse events or side effects during the postoperative period.

### Statistical analysis

The results obtained from the study are presented in a tabulated manner. The results are expressed as mean [standard deviation (SD)]. Comparisons between groups were performed with the Kruskal-Wallis one-way analysis of variance (ANOVA) by ranks or Fisher's exact test for small samples with a 5% risk. Mann-Whitney-Wilcoxon tests were performed when normality tests failed (Graph Pad InStat version 3.05, Graph Pad Software, SanDiego, CA, USA)

## RESULTS

All the three groups were comparable with respect to age, sex, body weight and the duration of surgery (*P* > 0.05) [Table 1]. The time for BIS to reach 60 was considerably longer in Group P compared to Group C or Group M (*P* < 0.0001). In recovery period, the time to reach BIS to 80 was found to be significantly less in Group C compared to Group M and Group P [*P* < 0.0001] [Table 2]. Requirement of propofol was found to be significantly lower in Group C and Group M compared to Group P, both for induction of anaesthesia and for maintenance (*P* < 0.001) [Table 3]. Fentanyl requirement was also found to be lower in Group C and Group M, compared to Group P (*P* < 0.001) [Table 3]. When the groups were compared, all the three parameters of recovery (extubation time, response to verbal commands and time for orientation) were found to be significantly longer in Group M. There were no significant differences between the other two groups, i.e. Group C and Group P (*P* > 0.05) [Table 4].

**Table 1:** Patient characteristics given as mean (SD)

	Group C	Group M	Group P	*P* value
Age (years)	37.96	43.52	35.8	0.07
	(9.61)	(8.52)	(9.51)	
Sex (male/female)	20/5	19/6	14/11	0.73
Weight (kg)	62.48	66.92	64.88	0.47
	(10.76)	(10.78)	(10.8)	
Duration of	109.2	111.28	116.32	0.58
surgery (minutes)	(9.48)	(13.3)	(11.56)	

**Table 2:** Induction (BIS < 60) and recovery (BIS > 80) time in different groups given as mean (SD)

Induction and recovery time	Group C	Group M	Group P	*P* value
Induction period	54.36	59.6	80.28	0.0000
BIS < 60 (seconds)	(7.42)*	(7.47)*	(16.64)	
Recovery period	5.8	8.92	6.78	0.0000
BIS > 80 (minutes)	(1.19)*	(2.85)	(1.51)*	

* Significant differences within groups (*P* < 0.0001)

**Table 3:** Requirement of propofol and fentanyl during anaesthesia given as mean (SD)

Requirement of anaesthetic agents	Group C	Group M	Group P	*P* value
Propofol induction	1.62	1.65	2.24	0.000
(mg/kg)	(0.26)*	(0.23)*	(0.5)	
Propofol	3.92	4.58	6.54	0.000
maintenance (mg/kg/hour)	(0.68)*	(0.85)*	(1.44)	
Fentanyl (μg/kg)	1.12	1.33	2.54	0.000
	(0.26)*	(0.29)*	(0.94)	

* Significant differences within groups (*P* < 0.001)

**Table 4:** Recovery time following discontinuation of all the infusions

Parameters to measure recovery time	Group C	Group M	Group P	*P* value
Extubation time	5.43	8.48	6.62	0.000
(minutes)	(0.8)	(2.17)*	(1.48)	
Response to	7.88	9.99	7.84	0.000
verbal command (minutes)	(1.69)	(2.24)*	(1.68)	
Time for	9.53	10.44	8.82	0.000
orientation (minutes)	(1.41)	(1.48)*	(1.07)	

* Significant differences within groups (*P* < 0.001)

MAP values in Group P were significantly higher (*P* < 0.05) after intubation compared with preoperative value [Table 5]. MAP in Group M decreased significantly after induction (*P* < 0.001) [Table 5]. MAP in Group C decreased significantly for all measurements with the exception of intubation and after infusion (*P* < 0.001) [Table 5]. Heart rate in Group P increased significantly after intubation and extubation (*P* < 0.01) but remained comparable to the preoperative value (*P* > 0.05) during rest of the period [Table 6]. Heart rate in Group C decreased significantly in all periods with the exception of postinfusion, postintubation and extubation (*P* < 0.001) [Table 6]. Heart rate in Group M was significantly lower after induction (*P* < 0.05) and in the intraoperative period (*P* < 0.05) except in postintubation period [Table 6].

**Table 5:** Mean arterial pressure given as mean (SD)

MAP	Group C	Group M	Group P
Preoperative	106.4 (13)	103 (11.3)	104 (10.7)
After infusion	87 (16.7)	90.1 (19)	106.2 (13.3)
1 minute after induction	60 (16.8)***	66 (16.7)***	92 (25.4)
1 minute after intubation	88.5 (25.1)	98.2 (25.4)	118.2 (16.3)*
10 minutes	80.3 (13.6)*	90 (17.4)*	100 (15.7)
30 minutes	80.3 (13.9)*	90 (16.3)*	97.2 (16)
60 minutes	81.1 (13.8)*	91.2 (16.2)*	98 (15.4)
90 minutes	78.2 (13.2)**	92.1 (15.9)*	100.1 (13.3)
120 minutes	80.2 (12.2)*	88.2 (16.5)*	102 (10.9)

**Table 6:** Heart rate given as mean (SD)

Heart rate	Group C	Group M	Group P
Preoperative	78 (12.2)	80 (11.5)	79.1 (10)
After infusion	74.1 (11.4)	80 (11.4)	82 (9.6)
1 minute after induction	66.2 (16.1)***	70.1 (16.7)*	79.4 (13)
1 minute after intubation	79.1 (12)	82 (9.6)	98.2 (14.7)**
10 minutes	62.4 (12.4)***	64.1 (13.6)*	88.1 (17.9)**
30 minutes	60.1 (9.8)***	65.2 (12.8)*	80.3 (10.8)
60 minutes	58.2 (7.3)***	62.2 (11.4)*	80 (11.5)
90 minutes	64.1 (13.6)***	60.1 (9.8)*	80.4 (11.2)
120 minutes	64.6 (12.5)***	68.1 (11.8)*	78 (12.4)
Postoperative period	76 (11.2)	82.2 (9.8)	90.2 (16.8)**
Postoperative period	86.1 (7.6)*	98 (15.5)	110.1 (18.7)

* *P* < 0.05,

** *P* < 0.01,

*** *P* < 0.001 compared with preoperative period

## DISCUSSION

In our study, we observed the effects of clonidine and MgSO_4_ as adjuvants of general anaesthesia. Our results demonstrate a significant reduction in consumption of propofol and fentanyl used for balanced anaesthesia with both clonidine and MgSO_4_. Importantly, we used an objective, qualitative measure of anaesthetic state (BIS) to guide anaesthetic requirements and to determine endpoints.[8‐11]

Altan and Turgut[12] used clonidine 3 *μ*g/kg intravenously over a period of 15 minutes before induction and 2 μg/kg/hour by continuous infusion intraoperatively. They observed significant incidences of bradycardia and hypotension in their study.[12] Kulka and Tryba[13] observed similar incidences of bradycardia and hypotension in their study.[13] Based on these observations, we administered clonidine 3 μg/kg intravenously 15 minutes before induction and reduced the infusion to 1 μg/kg/hour intraoperatively in our study. In spite of this reduced infusion rate of clonidine, we observed significant incidences of bradycardia and hypotension in our study. Further studies using lesser dose of clonidine may be necessary.

Elsharnouby and Elsharnouby[14] used MgSO_4_ 40 mg/kg intravenously over a period of 15 minutes before induction and 15 mg/kg/hour by continuous infusion intraoperatively. They noticed more episodes of severe hypotension using this dose of MgSO_4_. In our study, we reduced the dose of MgSO_4_ to30 mg/kg before induction and 10 mg/kg/hour by continuous infusion intraoperatively. The dose selected by us resulted in a steady and smooth reduction of MAP and heart rate, with no episodes of severe hypotension and bradycardia. Our finding was supported by a study conducted by Telci and Esen,[15] who used similar dose of MgSO_4_ as ours.

In our study, propofol and fentanyl requirements were significantly lower in patients of both Group C and Group M in comparison to Group P. Studies with rat model showed that at clinical concentration, clonidine partially inhibits voltage-gated Na and K channels and suppresses the generation of action potentials in tonic firing spinal dorsal horn neurons.[13] This may contribute to the reduction of propofol and fentanyl requirements. Fehr and Zalunardo[16] observed similar findings in their study.

MgSO_4_ has been reported to produce general anaesthesia and enhance the activity of local anaesthetic agents.[2] Depressant effects of MgSO_4_ on the central nervous system (CNS) of animals has been reported too.[3] Magnesium antagonised NMDA receptors in the CNS.[4] Another mechanism could involve the reduction of catecholamine release through sympathetic stimulation by which magnesium might decrease peripheral nociceptor sensitisation or stress response to surgery. However, these mechanisms do not explain the reduction in propofol requirements, independent of the reduction of the requirement of fentanyl. Clearly, further studies on the interaction between magnesium and propofol as sole agents need to be done. By acting as an antagonist of NMDA receptors, magnesium has the potential to prevent pain. The effect of magnesium on perioperative analgesic requirement was first evaluated by Koinig and colleagues[6] in patients with identical level of surgical stimulation. This is also confirmed in a study done by Shulz-Stubher *et al*.[17]

Taittoven and colleagues[18] compared clonidine and midazolam as premedication agents and observed no differences in oxygen consumption, anxiolysis, energy expenditures and CO_2_ production. Administration of clonidine before induction and intraoperatively results in improved perioperative haemodynamic stability. Preoperative oral clonidine protects against the pressure response to intubation.[19] Hypotension and bradycardia have been encountered with clonidine.[13] Clonidine can provide better perioperative haemodynamic stability in patients with mild to moderate hypertension. In laparoscopic surgical procedures where adverse cardiovascular change like increased arterial pressure is common, haemodynamic effect like hypotension may actually be beneficial. Van Den Berg and colleagues[20] found that MgSO_4_ attenuated the haemodynamic response to endotracheal intubation. In our study, both clonidine and MgSO_4_ lowered the haemodynamic response to intubation but clonidine was more effective in attenuating the sympathetic response.

In our study, recovery time was significantly prolonged in patients receiving MgSO_4_ in comparison to other two groups. The delay in recovery may be due to CNS depressant effect of MgSO_4_. A narcotic state in human beings undergoing surgical operations was achieved in a study by Peck and Meltzer,[21] who attempted anaesthesia by MgSo_4_ infusion in three patients of herniorhaphy. However, Aldrete and Vazeery [22] suggested this was actually a sleep-like state caused by cerebral hypoxia from progressive respiratory and cardiac depression. When ventilation was maintained, even very high level of serum Mg produced no CNS depression.

To conclude, perioperative use of both clonidine and magnesium sulphate significantly reduced the requirement of propofol and fentanyl citrate. They were able to attenuate the haemodynamic response to tracheal intubation. Both clonidine and magnesium sulphate caused bradycardia and hypotension. Besides, magnesium sulphate caused a delay in recovery. Therefore, both clonidine and magnesium sulphate need careful management, to be used as adjuvant agents to general anaesthetics.
